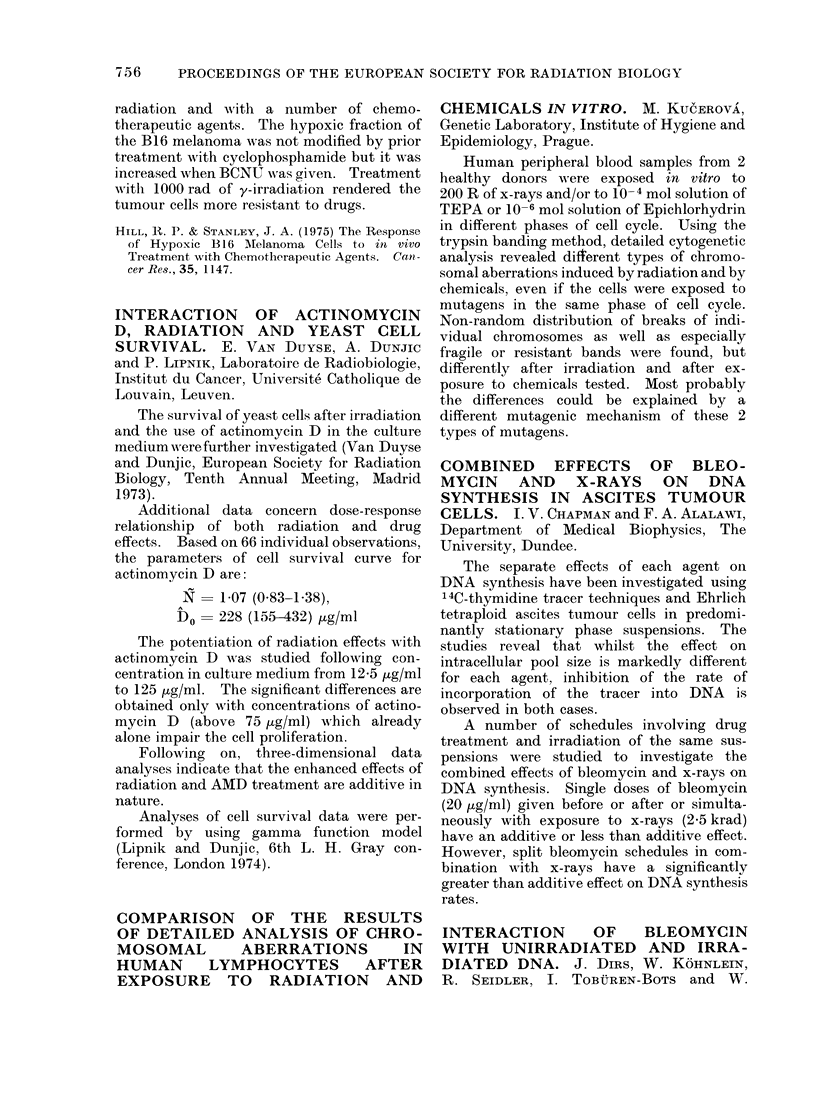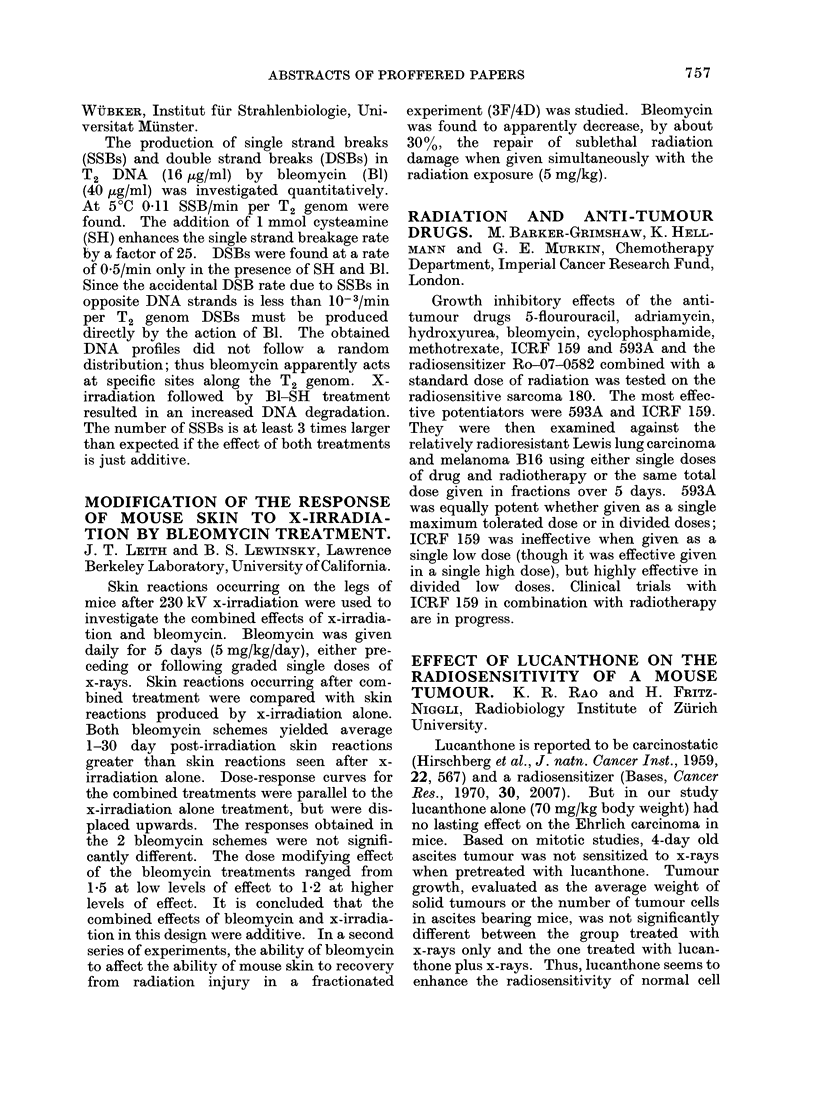# Proceedings: Interaction of bleomycin with unirradiated and irradiated DNA.

**DOI:** 10.1038/bjc.1975.307

**Published:** 1975-12

**Authors:** J. Dirs, W. Köhnlein, R. Seidler, I. Tobüren-Bots, W. Wübker


### INTERACTION OF BLEOMYCIN WITH UNIRRADIATED AND IRRADIATED DNA.

J. DIRS, W. KÖHNLEIN, R. SEIDLER, I. TOBÜREN-BOTS and W.

WÜBKER, Institut für Strahlenbiologie, Universitat Münster.

The production of single strand breaks (SSBs) and double strand breaks (DSBs) in $T_2$ DNA (16 $\mu$g/ml) by bleomycin (Bl) (40 $\mu$g/ml) was investigated quantitatively. At 5°C 0·11 SSB/min per $T_2$ genom were found. The addition of 1 mmol cysteamine (SH) enhances the single strand breakage rate by a factor of 25. DSBs were found at a rate of 0·5/min only in the presence of SH and Bl. Since the accidental DSB rate due to SSBs in opposite DNA strands is less than $10^{-3}$/min per $T_2$ genom DSBs must be produced directly by the action of Bl. The obtained DNA profiles did not follow a random distribution; thus bleomycin apparently acts at specific sites along the $T_2$ genom. X-irradiation followed by Bl–SH treatment resulted in an increased DNA degradation. The number of SSBs is at least 3 times larger than expected if the effect of both treatments is just additive.